# Enzalutamide Versus Abiraterone After Docetaxel in Metastatic Castration-Resistant Prostate Cancer: Real-World Outcomes and Exploratory Prognostic Stratification

**DOI:** 10.3390/jcm15124816

**Published:** 2026-06-21

**Authors:** Mert Tohumcuoğlu, Tolga Köşeci, Alpay Düşgün, Abdullah Evren Yetişir, Cem Mirili, Burak Mete, Mahmut Büyükşimşek

**Affiliations:** 1 Department of Medical Oncology, Adana City Training and Research Hospital, University of Health Sciences, Adana 01230, Türkiye; alpaydusgun@hotmail.com (A.D.); evrenyetisir@hotmail.com (A.E.Y.); mahmutbuyuksimsek@gmail.com (M.B.); 2 Department of Medical Oncology, Faculty of Medicine, Çukurova University, Adana 01330, Türkiye; drtolgakoseci@gmail.com; 3 Medical Oncology Clinic, Adana Ortadoğu Hospital, Adana 01140, Türkiye; cemirili@gmail.com; 4 Department of Public Health, Faculty of Medicine, Çukurova University, Adana 01330, Türkiye; burakmete2008@gmail.com

**Keywords:** metastatic castration-resistant prostate cancer, abiraterone acetate, enzalutamide, docetaxel, pan-immune-inflammation value, prognostic score, overall survival, nomogram

## Abstract

**Background/Objectives:** Enzalutamide and abiraterone acetate are commonly used androgen receptor pathway inhibitors in metastatic castration-resistant prostate cancer (mCRPC), including after docetaxel. However, real-world outcomes remain heterogeneous, and simple prognostic markers may help describe this variability. This study aimed to describe survival outcomes with enzalutamide and abiraterone acetate after docetaxel and to explore the prognostic value of a routine clinical-inflammatory risk classification. **Methods:** This retrospective single-center study included 136 patients with mCRPC treated with enzalutamide or abiraterone acetate after docetaxel. A composite risk classification was defined using four routinely available variables: pan-immune-inflammation value (PIV) > 457.99, time to castration resistance < 12 months, baseline hemoglobin ≤ 12 g/dL, and Gleason score ≥ 8. One point was assigned for each adverse factor, and patients were classified as low, moderate, or high risk. Overall survival (OS) was assessed using Kaplan–Meier estimates and Cox regression. The prognostic score and Cox regression-based nomogram were evaluated as exploratory tools. **Results:** Of the 136 patients, 8 (5.9%) were classified as low risk, 67 (49.3%) as moderate risk, and 61 (44.9%) as high risk. Median OS was not reached in the low-risk group, compared with 33.84 months in the moderate-risk group and 9.66 months in the high-risk group. In multivariable analysis, high-risk status was independently associated with worse OS (HR = 9.87; 95% CI: 2.38–40.92; *p* = 0.002). No statistically significant OS difference was observed between enzalutamide and abiraterone acetate in this non-randomized cohort (HR = 1.36; 95% CI: 0.90–2.06; *p* = 0.142). **Conclusions:** In this real-world post-docetaxel mCRPC cohort, no statistically significant OS difference was observed between enzalutamide and abiraterone acetate; however, the study was not designed to establish comparative effectiveness or therapeutic equivalence. The exploratory risk classification based on routine clinical and inflammatory variables was associated with distinct survival outcomes. External validation is required before clinical application.

## 1. Introduction

Prostate cancer remains one of the most commonly diagnosed malignancies worldwide and continues to account for a substantial proportion of cancer-related mortality. According to GLOBOCAN 2022, it was responsible for approximately 1.47 million new cases and nearly 400,000 deaths globally, underscoring the persistent burden of advanced disease [1]. For many patients, the development of metastatic castration-resistant prostate cancer (mCRPC) represents an important clinical turning point, as progression despite androgen deprivation is associated with treatment resistance, increasing metastatic burden, and reduced survival [2].

Docetaxel was the first systemic treatment to demonstrate a clear survival benefit in mCRPC and remains an important milestone in the management of advanced prostate cancer [3]. The therapeutic landscape subsequently expanded with the introduction of androgen receptor pathway inhibitors, including abiraterone acetate and enzalutamide. In the post-docetaxel mCRPC setting, both agents improved overall survival and became widely used life-prolonging treatment options [4,5,6]. However, choosing between these agents in routine practice remains difficult. European guidelines recognize both abiraterone acetate and enzalutamide as appropriate treatment options, but the preferred agent for an individual patient is not always clear, particularly when comorbidities, disease tempo, prior treatment exposure, steroid suitability, toxicity concerns, and sequencing issues are considered [7]. In addition, no definitive phase III head-to-head trial has established the superiority of one agent over the other in this setting. Although several studies have compared abiraterone acetate and enzalutamide in different mCRPC populations, real-world evidence specifically focused on the post-docetaxel setting remains limited and inconsistent [8,9,10,11].

The clinical relevance of this question should also be interpreted in the context of a changing treatment landscape. Molecular stratification, PARP inhibitor-based strategies, PSMA-targeted treatments, and earlier use of androgen receptor pathway inhibitors have increasingly influenced the management of metastatic prostate cancer [7]. Recent evidence in metastatic hormone-sensitive prostate cancer has also emphasized the clinical importance of visceral disease as a high-risk feature, reinforcing the need to interpret outcomes in metastatic prostate cancer according to baseline disease characteristics [12]. Nevertheless, in many real-world settings, patients who remain androgen receptor pathway inhibitor-naïve after docetaxel still receive abiraterone acetate or enzalutamide for mCRPC. Therefore, outcome data from this treatment sequence may still be clinically useful, particularly when viewed as pragmatic real-world evidence rather than as a definitive guide to modern treatment sequencing.

A further challenge in mCRPC is the marked heterogeneity of prognosis. Outcomes are influenced not only by treatment selection, but also by metastatic distribution, tumor burden, performance status, disease kinetics, and routine laboratory parameters. Several prognostic models developed in the docetaxel and post-docetaxel eras have emphasized the importance of these variables [13,14,15,16]. Visceral metastasis has been consistently associated with poorer survival compared with bone-only or lymph-node-only disease [16]. Other readily available variables also carry prognostic information. A shorter time to castration resistance generally reflects a more aggressive disease course [17], whereas low hemoglobin may indicate advanced tumor burden, impaired systemic reserve, chronic inflammation, or bone marrow involvement and has repeatedly been associated with adverse outcomes [13,18]. Conventional clinicopathologic factors, including Gleason score and disease burden, also remain relevant in advanced prostate cancer [19]. Systemic inflammation has also gained attention as a practical marker of prognosis. Inflammation-based indices may reflect the interaction between tumor-promoting myeloid activity, platelet-mediated tumor progression, and lymphocyte-dependent antitumor immune response. The pan-immune-inflammation value (PIV) is a composite inflammatory marker derived from neutrophil, platelet, monocyte, and lymphocyte counts [20]. Its main advantage is that it can be calculated from routine complete blood counts without additional testing. This is particularly relevant in mCRPC, where patients are often older, heavily pretreated, and clinically heterogeneous. In patients with mCRPC treated with abiraterone acetate or enzalutamide, elevated PIV has been associated with shorter overall survival, suggesting that it may add prognostic information in androgen receptor-targeted treatment settings [21].

In this context, we described real-world survival outcomes among patients treated with enzalutamide or abiraterone acetate after docetaxel and examined whether a simple risk classification based on PIV, TTCR, hemoglobin, and Gleason score could help characterize prognostic heterogeneity. The treatment comparison was interpreted as observational and exploratory, rather than as a definitive comparative effectiveness analysis. An exploratory nomogram was also generated to visualize 12-month mortality risk, without intending it as a validated decision-making tool.

## 2. Materials and Methods

### 2.1. Study Design and Site

This retrospective cohort study was conducted at the Department of Medical Oncology, Adana City Training and Research Hospital, Adana, Türkiye. The study included patients with metastatic castration-resistant prostate cancer (mCRPC) who received enzalutamide or abiraterone acetate after prior docetaxel treatment.

### 2.2. Patient Selection and Study Population

Patients with mCRPC who had received docetaxel before second-line androgen receptor pathway inhibitor (ARPi) therapy with either enzalutamide or abiraterone acetate were retrospectively identified from the institutional electronic health record system and patient files. Patients who initiated second-line ARPi therapy between April 2018 and November 2024 were screened for eligibility. Follow-up data were updated through 24 December 2025. After applying the inclusion and exclusion criteria, the final analytic cohort consisted of 136 patients.

#### 2.2.1. Inclusion Criteria

Patients were eligible for inclusion if they met the following criteria: age ≥ 18 years; histopathologically confirmed prostate adenocarcinoma; diagnosis of mCRPC; previous treatment with docetaxel before second-line ARPi therapy; receipt of second-line treatment with enzalutamide or abiraterone acetate; and availability of the required clinical, laboratory, treatment, and follow-up data.

#### 2.2.2. Exclusion Criteria

Patients were excluded if they were younger than 18 years, lacked histopathological confirmation of prostate adenocarcinoma, had incomplete or insufficient clinical, laboratory, treatment, or follow-up data, had no follow-up information after ARPi initiation, had inconsistencies or errors in diagnosis, treatment sequence, or follow-up records after detailed review, or received a second-line systemic treatment other than enzalutamide or abiraterone acetate.

### 2.3. Clinical and Demographic Data Collection

Clinical, demographic, laboratory, pathological, treatment, and follow-up data were retrieved from the institutional electronic health record system and patient files. The collected variables included age at ARPi initiation, ARPi agent, Gleason pattern, Gleason score, Grade Group, metastatic sites, complete blood count parameters, hemoglobin level, alkaline phosphatase, lactate dehydrogenase, albumin, date of androgen deprivation therapy (ADT) initiation, date of castration-resistant disease development, date of ARPi initiation, baseline prostate-specific antigen (PSA), PSA50 response at 12 weeks, radiologic best response, progression status, vital status, and event or censoring dates for overall survival analysis. Metastatic involvement was recorded by site and included bone metastases, bone marrow involvement, distant non-regional lymph node metastases, liver metastases, lung metastases, other visceral metastases, and central nervous system metastases. Visceral metastasis was defined as the presence of liver, lung, other visceral organ, or central nervous system metastases.

### 2.4. Histopathological and Disease Assessment

Histopathological data were obtained from pathology reports and included Gleason pattern, Gleason score, and Grade Group. Disease extent was assessed using available radiologic and clinical records. Metastatic sites were categorized as bone, bone marrow, distant non-regional lymph node, liver, lung, other visceral organ, and central nervous system metastases. Castration-resistant prostate cancer was defined as biochemical, radiologic, or clinical progression despite ongoing ADT and castrate serum testosterone levels < 50 ng/dL. Time to castration resistance (TTCR) was calculated as the interval between the date of ADT initiation and the date of CRPC development.

### 2.5. Treatment Protocol

All patients continued ADT to maintain castrate serum testosterone levels during treatment. Docetaxel had been administered before second-line ARPi therapy, generally at a dose of 75 mg/m^2^ every 3 weeks with oral prednisone or prednisolone, according to institutional practice and patient tolerance. After progression during or after docetaxel treatment and confirmation of castration-resistant disease, patients received second-line ARPi therapy with either enzalutamide or abiraterone acetate. Enzalutamide was administered orally at a dose of 160 mg once daily. Abiraterone acetate was administered orally at a dose of 1000 mg once daily in combination with prednisone or prednisolone 5 mg twice daily. Treatment was continued until disease progression, unacceptable toxicity, death, or physician discretion.

### 2.6. Calculation of Pan-Immune-Inflammation Value and Laboratory Parameters

Laboratory parameters were obtained before initiation of second-line ARPi therapy. Complete blood count parameters included neutrophil, monocyte, platelet, and lymphocyte counts. The pan-immune-inflammation value (PIV) was calculated as follows:

PIV = neutrophil count × platelet count × monocyte count/lymphocyte count

Hemoglobin level was recorded at the same time point. Serum alkaline phosphatase, lactate dehydrogenase, and albumin values obtained at the same baseline time point were also recorded for supplementary prognostic analyses. The cohort median PIV was 457.99. Therefore, PIVs above 457.99 were considered adverse for the exploratory risk classification.

### 2.7. Definition of the Exploratory Clinical-Inflammatory Risk Classification

An exploratory clinical-inflammatory risk classification was defined using routinely available clinical and laboratory variables reflecting systemic inflammation, disease kinetics, anemia, and tumor grade. The variables included in the classification were selected according to clinical relevance, biological plausibility, and routine availability in daily practice.

Cutoff values were selected according to clinical interpretability and cohort distribution. The median value was used for PIV, whereas TTCR < 12 months, baseline hemoglobin ≤ 12 g/dL, and Gleason score ≥ 8 were used as clinically relevant adverse thresholds. Equal weighting was chosen to keep the score simple and clinically interpretable; this approach was considered exploratory.

One point was assigned for each of the following adverse factors:

PIV > 457.99: 1 point;
TTCR < 12 months: 1 point;
Baseline hemoglobin ≤ 12 g/dL: 1 point;
Gleason score ≥ 8: 1 point.

The total score ranged from 0 to 4. Patients were then classified into three groups: low risk, 0 points; moderate risk, 1–2 points; and high risk, 3–4 points. This exploratory classification was used to evaluate overall survival, PSA50 response, and radiologic best response in patients receiving second-line ARPi therapy after docetaxel.

### 2.8. Exploratory Nomogram Development

A Cox regression-based nomogram was generated only as an exploratory visual summary of 12-month mortality risk. Candidate variables were selected according to clinical relevance, routine availability, and biological plausibility in mCRPC rather than through automated variable selection. The model included Gleason score, PIV, hemoglobin level, TTCR, and the number of metastatic sites. The number of metastatic sites was calculated by summing the documented metastatic site categories for each patient. Because the nomogram was derived from the same retrospective cohort and was not externally validated, calibrated in an independent dataset, or assessed by decision-curve analysis, it was not intended for clinical decision-making.

### 2.9. Follow-Up and Outcome Assessment

Patients were followed according to institutional routine clinical practice. Because this was a retrospective real-world study, follow-up intervals and imaging schedules were not fully standardized across all patients. Follow-up information was obtained from the institutional electronic health record system and patient files. The primary endpoint was overall survival (OS), defined as the time from initiation of second-line ARPi therapy to death from any cause or last follow-up. Patients who were alive at the last follow-up were censored. Progression status was recorded when radiologic progression was documented during follow-up. However, progression-free survival was not analyzed as a time-to-event endpoint because imaging intervals were not fully standardized in this retrospective cohort. PSA50 response was defined as a ≥50% decline in PSA from baseline at 12 weeks after ARPi initiation. Radiologic best response was determined according to available imaging reports and categorized based on the best documented response during treatment.

### 2.10. Statistical Analysis

Statistical analyses were performed using jamovi software, version 2.6.44. Normality of continuous variables was assessed using the Shapiro–Wilk test. Continuous variables were presented as mean ± standard deviation or median with interquartile range, according to distribution. Categorical variables were presented as frequencies and percentages. Comparisons between groups were performed using the Mann–Whitney U test or Kruskal–Wallis test for continuous variables, as appropriate. Categorical variables were compared using the chi-square test or Fisher’s exact test, depending on expected cell counts. Survival curves for overall survival were estimated using the Kaplan–Meier method and compared with the log-rank test. Median follow-up was estimated using the reverse Kaplan–Meier method. Cox proportional hazards regression analysis was used to evaluate factors associated with overall survival. Univariable Cox regression analyses were first performed. The main multivariable Cox regression model included risk classification, ARPi agent, and age. As supplementary analyses, baseline characteristics were compared according to ARPi agent, an extended multivariable Cox model was performed using available baseline prognostic variables, and the individual components of the risk score were evaluated separately. Skewed continuous variables, including PSA, alkaline phosphatase, and lactate dehydrogenase, were log-transformed for the extended Cox model. The proportional hazards assumption was evaluated using Schoenfeld residuals where applicable. Model discrimination was assessed using Harrell’s C-index. A two-sided *p* value < 0.05 was considered statistically significant. Patients with missing or incomplete required clinical, laboratory, treatment, or follow-up data were excluded from the final analytic cohort; therefore, complete-case analysis was used. No imputation was performed.

### 2.11. Ethics Committee Approval

The study protocol was reviewed and approved by the Scientific Research Ethics Committee of Adana City Training and Research Hospital, Adana, Türkiye. The committee approved the retrospective study entitled “Comparison of the Efficacy of Abiraterone and Enzalutamide as Second-Line Treatment After Docetaxel in Metastatic Castration-Resistant Prostate Cancer” with Meeting No: 17, Date: 25 September 2025, and Decision No: 761. The requirement for written informed consent was waived because of the retrospective design of the study and the use of anonymized patient data.

## 3. Results

### 3.1. Baseline Characteristics, Disease Distribution, and Treatment Response Across Risk Groups

A total of 136 patients with metastatic castration-resistant prostate cancer who received abiraterone acetate or enzalutamide after docetaxel were included in the analysis. According to the risk score, 8 patients (5.9%) were classified as low risk, 67 patients (49.3%) as moderate risk, and 61 patients (44.9%) as high risk. Age did not differ significantly among the risk groups (*p* = 0.795). The distribution of ARPi treatment was also comparable across groups, with no significant imbalance between abiraterone acetate and enzalutamide use (*p* = 0.549). A separate baseline comparison according to ARPi agent is provided in Supplementary Table S1. Most clinical and disease-related variables were not significantly different between the abiraterone acetate and enzalutamide groups, although PIV was higher in patients treated with enzalutamide. Gleason score differed significantly among the groups and was higher in the high-risk group (*p* < 0.001). Regarding metastatic disease distribution, distant non-regional lymph node metastases and liver metastases differed significantly among the risk groups (*p* = 0.036 and *p* = 0.014, respectively). Bone metastases, bone marrow involvement, lung metastases, other visceral metastases, and central nervous system metastases did not show statistically significant differences across the groups. Serum PSA levels differed significantly among the risk groups and were highest in the high-risk group (*p* < 0.001). PSA50 response at 12 weeks was less frequent in the high-risk group (*p* < 0.001). Radiologic best response also differed significantly among the groups (*p* = 0.009). In addition, TTCR < 12 months was significantly more frequent in the high-risk group (*p* < 0.001) (Table 1).

### 3.2. Overall Survival According to Risk Groups

**Figure 1:**
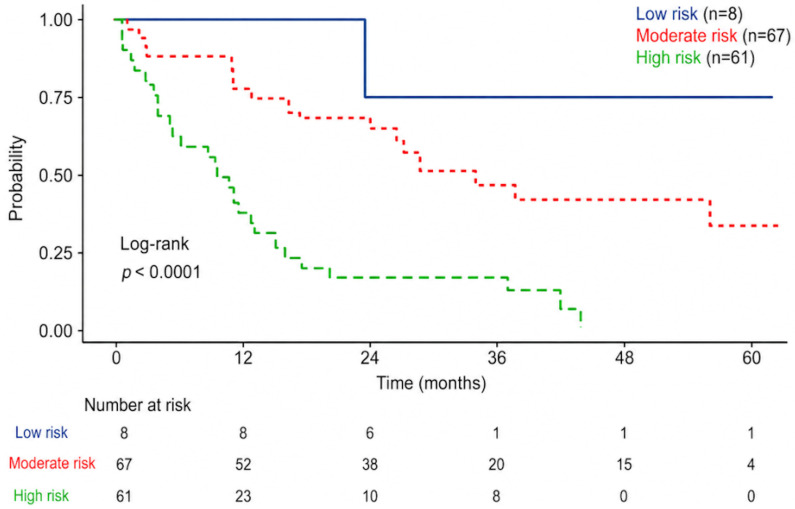
Kaplan–Meier overall survival curves according to the exploratory risk groups.

Kaplan–Meier analysis showed separation in overall survival according to risk group (Figure 1). The survival analysis included 136 patients and 95 death events. Median follow-up, estimated by the reverse Kaplan–Meier method, was 40.5 months. Median overall survival was not reached in the low-risk group, whereas it was 33.84 months in the moderate-risk group and 9.66 months in the high-risk group. The risk score showed moderate discriminatory ability for overall survival, with a C-index of 0.674.

In univariable Cox regression analysis, the moderate-risk group had a numerically higher risk of death than the low-risk group, although this difference was not statistically significant (HR = 2.53; 95% CI: 0.61–10.54; *p* = 0.201). The high-risk group had a significantly higher mortality risk compared with the low-risk group (HR = 9.15; 95% CI: 2.22–37.72; *p* = 0.002) (Table 2).

Pairwise survival comparisons showed that the high-risk group had significantly worse overall survival than both the low-risk and moderate-risk groups (both *p* < 0.001). The difference between the low-risk and moderate-risk groups was not statistically significant (*p* = 0.158). Survival estimates decreased across the risk groups. One-year overall survival was 100.0% in the low-risk group, 77.6% in the moderate-risk group, and 37.7% in the high-risk group. The corresponding 36- and 60-month estimates are provided in Table 3, together with the number of patients at risk at each time point.

### 3.3. Multivariable Cox Regression Analysis for Overall Survival

In the main multivariable Cox regression model adjusted for age and ARPi agent, the risk score remained associated with overall survival. Compared with the low-risk group, the moderate-risk group showed a non-significant increase in mortality risk (HR = 2.62; 95% CI: 0.63–10.92; *p* = 0.185), whereas the high-risk group remained independently associated with worse overall survival (HR = 9.87; 95% CI: 2.38–40.92; *p* = 0.002). ARPi agent was not significantly associated with overall survival in this model (enzalutamide vs. abiraterone acetate: HR = 1.36; 95% CI: 0.90–2.06; *p* = 0.142). The model showed modest discrimination, with a C-index of 0.686 (Table 4). As a supplementary analysis, an extended multivariable Cox model was performed using available baseline prognostic variables. In this model, ARPi agent remained not significantly associated with overall survival, whereas hemoglobin, alkaline phosphatase, lactate dehydrogenase, albumin, and Gleason score were associated with overall survival; model discrimination was higher than in the main model (C-index = 0.830) (Supplementary Table S2). The individual components of the exploratory risk score were also examined separately and are reported in Supplementary Table S3.

### 3.4. Exploratory Nomogram for 12-Month Mortality Risk

**Figure 2:**
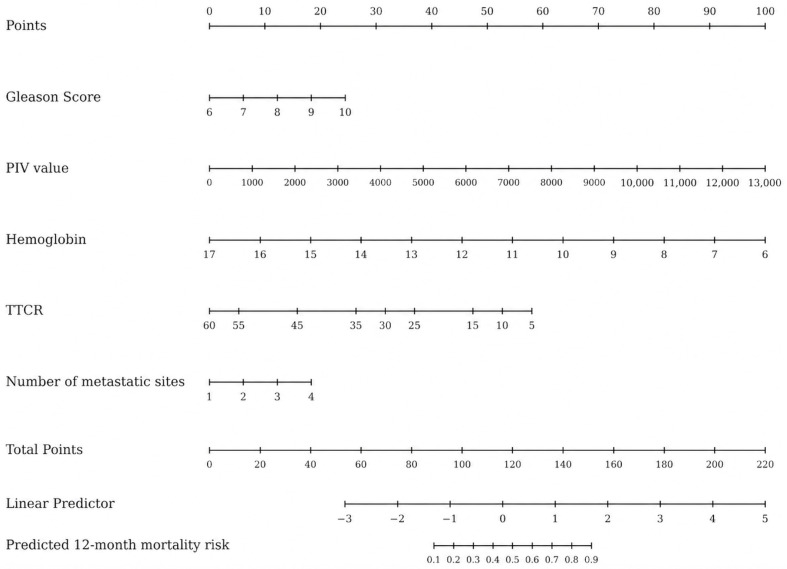
Cox regression-based exploratory nomogram for estimating individualized 12-month mortality risk.

A Cox regression-based nomogram was generated as an exploratory visual model for 12-month mortality risk. It included Gleason score, PIV, hemoglobin level, TTCR, and number of metastatic sites. Higher Gleason score, higher PIV, lower hemoglobin level, shorter TTCR, and a greater number of metastatic sites contributed to higher total points. Because the model was derived from the same retrospective cohort and was not externally validated, the nomogram should be interpreted only as a descriptive, hypothesis-generating tool rather than as a clinical prediction model (Figure 2).

## 4. Discussion

In this retrospective real-world cohort of patients with mCRPC treated with abiraterone acetate or enzalutamide after docetaxel, ARPi agent was not significantly associated with overall survival after adjustment for age and risk group. This finding should be interpreted cautiously because treatment allocation was not randomized and the study was not designed to establish comparative effectiveness. In contrast, the exploratory risk score based on PIV, TTCR, hemoglobin, and Gleason score separated patients into groups with different survival outcomes. Patients in the high-risk group had shorter overall survival, lower PSA50 response rates, and less favorable radiologic response patterns. The nomogram should therefore be viewed only as a descriptive exploratory tool.

The absence of a statistically significant overall survival difference between abiraterone acetate and enzalutamide requires careful interpretation. Both agents have demonstrated survival benefit in the post-docetaxel mCRPC setting, but no definitive phase III head-to-head trial has established the superiority of one agent over the other [4,5,6]. Real-world comparisons have also reported variable findings, probably because treatment selection is influenced by comorbidities, disease tempo, prior treatment exposure, physician preference, steroid suitability, toxicity concerns, and access-related factors [8,9,10,11]. Horváth et al. reported longer overall survival with enzalutamide than with abiraterone in a real-world comparison including first- and second-line mCRPC settings, and Aprikian et al. reached a similar overall conclusion in a meta-analysis of real-world studies [22,23]. However, such findings are not uniform across smaller institutional cohorts, and residual confounding remains an important limitation of non-randomized comparisons. For this reason, the present finding should not be read as evidence that the two agents are equivalent or interchangeable. It only indicates that, within this retrospective cohort, no statistically significant OS difference was detected after the available adjustments. Baseline prognostic status appeared to explain more of the survival heterogeneity than the choice between the two ARPi agents.

The differences in survival across risk groups are also consistent with the biological relevance of the variables included in the score. The score combines four routinely available variables that reflect different aspects of aggressive mCRPC: systemic inflammation, disease kinetics, anemia, and tumor grade. High PIV may indicate a systemic inflammatory profile driven by neutrophils, platelets, and monocytes, together with relative impairment of lymphocyte-mediated antitumor activity [20,21]. Yazgan et al. reported the prognostic value of PIV in patients with mCRPC treated with androgen receptor-signaling inhibitors, and later data from the same group suggested a similar prognostic role in patients receiving lutetium-177–PSMA-617 [21,24]. Short TTCR is consistent with early endocrine resistance and more aggressive disease behavior [17]. Low hemoglobin may reflect advanced tumor burden, impaired systemic reserve, marrow involvement, chronic inflammation, or treatment-related frailty [13,18]. Gleason score, although originally defined at diagnosis, remains one of the basic markers of tumor aggressiveness in prostate cancer [19]. Overall, these variables appear to reflect both tumor-related aggressiveness and host-related vulnerability. When the individual score components were examined separately, their associations with survival were not uniform. TTCR, hemoglobin, and Gleason score showed stronger individual associations, whereas dichotomized PIV did not retain a clear association in this cohort. This supports the view that the score should be interpreted as a simple exploratory classification rather than as a weighted prognostic model.

Survival outcomes followed the same pattern. Median overall survival was not reached in the low-risk group, whereas it was 33.84 months in the moderate-risk group and 9.66 months in the high-risk group. The later survival estimates, especially in the low-risk group, should be interpreted with caution because few patients remained at risk during longer follow-up. In multivariable analysis, the high-risk group remained independently associated with worse overall survival. The moderate-risk group showed an intermediate but statistically non-significant increase in mortality risk, which may partly reflect the heterogeneity of this category. Patients in the moderate-risk group had one or two adverse factors, whereas the high-risk group represented a more concentrated adverse phenotype.

Earlier prognostic models reported by Halabi et al., Chi et al., and Armstrong et al. identified variables such as performance status, hemoglobin, alkaline phosphatase, lactate dehydrogenase, PSA-related parameters, metastatic distribution, and treatment history as important determinants of survival in mCRPC [13,14,15]. The meta-analysis by Halabi et al. also emphasized the prognostic importance of metastatic site, particularly visceral involvement [16]. Compared with these more complex models, the present score relies on a small number of variables that are already available in routine care. It does not require molecular testing, advanced imaging metrics, or specialized assays.

The nomogram was included only to visualize how routinely available variables may contribute to estimated 12-month mortality risk. Kawahara et al. previously developed and validated a survival nomogram and calculator for patients with mCRPC treated with abiraterone and/or enzalutamide, showing that individualized prognostic prediction is feasible in this setting [25]. The present nomogram, however, was developed within a small retrospective cohort and was not externally validated. Calibration, optimism-corrected discrimination, and decision-curve analysis were also not performed. Therefore, it should not be considered a clinical prediction tool at this stage.

From a clinical perspective, these findings underline the importance of baseline prognostic assessment in patients receiving ARPi therapy after docetaxel. In routine practice, the question is often framed as whether abiraterone acetate or enzalutamide should be preferred. Although this remains clinically relevant, the present data suggest that readily available prognostic variables may help describe survival heterogeneity in this setting. If validated, such an approach may support risk-adapted follow-up, but it should not be used to guide treatment selection on the basis of the present study alone.

This study has several limitations. Its retrospective, single-center design creates the possibility of selection bias, information bias, and unmeasured confounding. Treatment allocation was not randomized, and the choice of abiraterone acetate or enzalutamide may have been influenced by clinical factors that were not fully captured in the dataset, including comorbidities, steroid suitability, disease tempo, prior docetaxel tolerance, physician preference, and access-related factors. Although an extended supplementary Cox model was added using available baseline variables, several established prognostic factors could not be fully incorporated, including ECOG performance status, detailed docetaxel exposure, response to docetaxel, and subsequent life-prolonging therapies. The low-risk group was small, which limited the precision of survival estimates and contributed to wide confidence intervals in comparisons involving this group. Follow-up intervals and imaging schedules were not fully standardized, reflecting the real-world nature of the cohort. The risk score was derived and evaluated in the same cohort, used a cohort-specific PIV cutoff, and assigned equal weight to all components. Propensity score matching or weighting was not performed because of the limited sample size and the exploratory nature of the treatment comparison. Spline-based analyses and bootstrap internal validation were also not performed. Together, these limitations reduce generalizability and support interpreting the score as descriptive rather than validated.

The clinical relevance of this treatment sequence should also be framed in the context of the changing metastatic prostate cancer landscape. ARPi agents are now increasingly used earlier in the metastatic hormone-sensitive setting, either with androgen deprivation therapy or as part of triplet strategies in selected patients. As a result, ARPi-naïve patients receiving abiraterone acetate or enzalutamide after docetaxel for mCRPC may become less common in contemporary practice. The present study should therefore not be interpreted as a recommendation for this sequence over newer treatment strategies. Rather, it provides real-world data from a clearly defined post-docetaxel cohort and explores whether routinely available variables can help describe prognostic heterogeneity within this setting. Future studies should validate these findings in more contemporary mCRPC cohorts, including patients who have received ARPi therapy earlier in the disease course.

## 5. Conclusions

In this retrospective real-world cohort of patients with mCRPC treated with abiraterone acetate or enzalutamide after docetaxel, the ARPi agent was not significantly associated with overall survival after adjustment for age and risk group. This finding should not be interpreted as evidence of therapeutic equivalence because treatment allocation was non-randomized and residual confounding remains possible. The exploratory risk score based on PIV, TTCR, hemoglobin, and Gleason score was associated with distinct survival outcomes. The high-risk group had shorter overall survival, lower PSA50 response rates, and less favorable radiologic response patterns. The nomogram should be regarded as hypothesis-generating only. Overall, these findings suggest that routinely available clinical and inflammatory variables may help describe prognostic heterogeneity in the post-docetaxel mCRPC setting. Further validation in larger, independent, and more contemporary cohorts is needed before clinical use.

## Figures and Tables

**Table 1:** Baseline Characteristics, Disease Distribution, and Treatment Response According to Risk Groups.

		Risk Score n (%) or Median (IQR)			
		Low	Moderate	High	*p* Value
Total n (%)		8 (5.9)	67 (49.3)	61 (44.9)	
Age	Median (IQR)	69.0 (65.8–74.2)	72.0 (65.5–73.5)	70.0 (62.0–75.0)	0.795
ARPi agent	Abiraterone	4 (50.0)	35 (52.2)	26 (42.6)	0.549
	Enzalutamide	4 (50.0)	32 (47.8)	35 (57.4)	
Gleason score	Median (IQR)	7.0 (7.0–7.0)	8.0 (7.0–9.0)	9.0 (8.0–9.0)	<0.001
Bone metastases	No	0 (0.0)	2 (3.0)	0 (0.0)	0.352
	Yes	8 (100.0)	65 (97.0)	61 (100.0)	
Bone marrow involvement	No	6 (75.0)	57 (85.1)	49 (80.3)	0.667
	Yes	2 (25.0)	10 (14.9)	12 (19.7)	
Distant (non-regional) lymph node metastases	No	3 (37.5)	47 (70.1)	31 (50.8)	0.036
	Yes	5 (62.5)	20 (29.9)	30 (49.2)	
Liver metastases	No	6 (75.0)	66 (98.5)	57 (93.4)	0.014
	Yes	2 (25.0)	1 (1.5)	4 (6.6)	
Lung metastases	No	8 (100.0)	53 (79.1)	51 (83.6)	0.322
	Yes	0 (0.0)	14 (20.9)	10 (16.4)	
Other visceral metastases	No	8 (100.0)	67 (100.0)	57 (93.4)	0.079
	Yes	0 (0.0)	0 (0.0)	4 (6.6)	
CNS metastases	No	8 (100.0)	67 (100.0)	59 (96.7)	0.287
	Yes	0 (0.0)	0 (0.0)	2 (3.3)	
Baseline PSA, ng/mL	Median (IQR)	7.2 (3.5–10.3)	12.9 (4.5–52.0)	65.7 (11.4–302.0)	<0.001
PSA50 at 12 weeks	No	0 (0.0)	14 (20.9)	37 (60.7)	<0.001
	Yes	8 (100.0)	53 (79.1)	24 (39.3)	
Radiologic best response	CR	0 (0.0)	2 (3.0)	0 (0.0)	0.009
	PR	8 (100.0)	48 (71.6)	32 (52.5)	
	SD	0 (0.0)	5 (7.5)	2 (3.3)	
	PD	0 (0.0)	12 (17.9)	27 (44.3)	
Time to castration resistance (TTCR)	≥12 months	8 (100.0)	45 (67.2)	12 (19.7)	<0.001
	<12 months	0 (0.0)	22 (32.8)	49 (80.3)	

Values are presented as n (%) or median (IQR). ARPi, androgen receptor pathway inhibitor; CNS, central nervous system; IQR, interquartile range; PSA, prostate-specific antigen; PSA50, ≥50% decline in PSA from baseline at 12 weeks; TTCR, time to castration resistance. CR, complete response; PR, partial response; SD, stable disease; PD, progressive disease.

**Table 2:** Univariable Cox Regression Analysis for Overall Survival According to Risk Groups.

Risk Group	Patients, n	Deaths, n	Median OS, Months	Univariable HR (95% CI)	*p* Value
Low	8	2	Not reached	Reference	—
Moderate	67	36	33.84	2.53 (0.61–10.54)	0.201
High	61	57	9.66	9.15 (2.22–37.72)	0.002

OS, overall survival; HR, hazard ratio; CI, confidence interval. Pairwise log-rank comparisons: Moderate vs. Low, *p* = 0.158; High vs. Low, *p* < 0.001; High vs. Moderate, *p* < 0.001. Model discrimination: C-index = 0.674.

**Table 3:** Overall Survival Rates According to Risk Groups.

Risk Group	Time Point, Months	Patients at Risk, n	Overall Survival, %	95% CI
Low	12	8	100.0	100.0–100.0
Low	36	1	75.0	50.3–100.0
Low	60	1	75.0	50.3–100.0
Moderate	12	52	77.6	68.2–88.3
Moderate	36	20	46.7	35.3–61.9
Moderate	60	4	33.7	21.6–52.4
High	12	23	37.7	27.3–52.1
High	36	8	16.4	9.3–28.9
High	60	0	0.0	NE

Values are Kaplan–Meier estimates and are presented at 12, 36, and 60 months. OS, overall survival; CI, confidence interval; NE, not estimable.

**Table 4:** Multivariable Cox Regression Analysis for Overall Survival.

Variable	Category/Value	Patients, n (%) or Mean (SD)	Univariable HR (95% CI)	*p* Value	Multivariable HR (95% CI)	*p* Value
ARPi agent	Abiraterone	65 (47.8)	Reference	-	Reference	-
	Enzalutamide	71 (52.2)	1.19 (0.79–1.78)	0.409	1.36 (0.90–2.06)	0.142
Risk group	Low	8 (5.9)	Reference	-	Reference	-
	Moderate	67 (49.3)	2.53 (0.61–10.54)	0.201	2.62 (0.63–10.92)	0.185
	High	61 (44.9)	9.15 (2.22–37.72)	0.002	9.87 (2.38–40.92)	0.002
Age	Per 1-year increase	69.0 (8.1)	1.00 (0.98–1.03)	0.950	0.99 (0.97–1.02)	0.565

Values are presented as n (%) unless otherwise indicated. ARPi, androgen receptor pathway inhibitor; CI, confidence interval; HR, hazard ratio; OS, overall survival. The multivariable model included ARPi agent, risk group, and age. Model discrimination: C-index = 0.686.

## Data Availability

The data supporting the findings of this study are not publicly available due to patient privacy, confidentiality, and ethical restrictions, and cannot be shared in accordance with institutional and ethical approval requirements.

## Supplementary Materials

The following supporting information can be downloaded at: https://www.mdpi.com/article/10.3390/jcm15124816/s1, Table S1: Baseline characteristics according to ARPi agent; Table S2: Extended multivariable Cox regression analysis for overall survival using available baseline prognostic variables; Table S3: Cox regression analysis of individual risk score components for overall survival.
