# Supplementary material for: Enzalutamide Versus Abiraterone After Docetaxel in Metastatic Castration-Resistant Prostate Cancer: Real-World Outcomes and Exploratory Prognostic Stratification
Source: J Clin Med. 2026 Jun 21;15(12):4816. doi: 10.3390/jcm15124816 (PMC13300754; doi:10.3390/jcm15124816)
Supplement: Supplementary file 1 [file jcm-15-04816-s001.zip › Supplementary Table S1.pdf]

Table S1. Baseline characteristics according to ARPi agent.

		ARPi agent n (%) or median (IQR)		
		**Abiraterone**	**Enzalutamide**	**p value**
Total n (%)		65 (47.8)	71 (52.2)	
Age	Median (IQR)	71.0 (65.0-75.0)	71.0 (62.0-74.5)	0.581
Gleason score	Median (IQR)	8.0 (7.0-9.0)	8.0 (7.0-9.0)	0.831
Gleason score ≥ 8	n (%)	46 (70.8)	47 (66.2)	0.567
Number of metastatic sites	Median (IQR)	2.0 (1.0-2.0)	2.0 (1.0-2.0)	0.131
Bone metastases	Yes	63 (96.9)	71 (100.0)	0.227
Bone marrow involvement	Yes	11 (16.9)	13 (18.3)	0.832
Distant non-regional lymph node metastases	Yes	22 (33.8)	33 (46.5)	0.134
Liver metastases	Yes	5 (7.7)	2 (2.8)	0.258
Lung metastases	Yes	9 (13.8)	15 (21.1)	0.266
Other visceral metastases	Yes	0 (0.0)	4 (5.6)	0.121
CNS metastases	Yes	0 (0.0)	2 (2.8)	0.497
Any visceral metastasis	Yes	14 (21.5)	17 (23.9)	0.738
Baseline PSA, ng/mL	Median (IQR)	23.2 (5.2-101.4)	27.2 (8.0-123.3)	0.419
Hemoglobin, g/dL	Median (IQR)	12.1 (10.7-13.1)	11.6 (10.8-12.8)	0.132
ALP, U/L	Median (IQR)	140.0 (73.0-218.0)	110.0 (86.5-185.0)	0.960
LDH, U/L	Median (IQR)	224.0 (193.0-333.0)	244.0 (209.0-371.5)	0.111
Albumin, g/dL	Median (IQR)	4.00 (3.50-4.10)	3.80 (3.30-4.10)	0.167
PIV	Median (IQR)	427.43 (205.50-641.13)	541.33 (371.37-909.75)	0.006
TTCR, months	Median (IQR)	11.99 (9.23-18.56)	10.58 (5.98-17.05)	0.105
TTCR <12 months	Yes	33 (50.8)	38 (53.5)	0.748
Hemoglobin ≤ 12 g/dL	Yes	31 (47.7)	42 (59.2)	0.181
Exploratory risk group	Low	4 (6.2)	4 (5.6)	0.549
	Moderate	35 (53.8)	32 (45.1)	
	High	26 (40.0)	35 (49.3)	

Values are presented as n (%) or median (IQR). p values were calculated using the Mann-Whitney U test for continuous variables and the chi-square or Fisher exact test for categorical variables, as appropriate. ARPi, androgen receptor pathway inhibitor; ALP, alkaline phosphatase; CNS, central nervous system; IQR, interquartile range; LDH, lactate dehydrogenase; PIV, pan-immune-inflammation value; PSA, prostate-specific antigen; TTCR, time to castration resistance.
