# Supplementary material for: Enzalutamide Versus Abiraterone After Docetaxel in Metastatic Castration-Resistant Prostate Cancer: Real-World Outcomes and Exploratory Prognostic Stratification
Source: J Clin Med. 2026 Jun 21;15(12):4816. doi: 10.3390/jcm15124816 (PMC13300754; doi:10.3390/jcm15124816)
Supplement: Supplementary file 1 [file jcm-15-04816-s001.zip › Supplementary Table S2.pdf]

Table S2. Extended multivariable Cox regression analysis for overall survival using available prognostic covariates.

Variable	Category / value	Multivariable HR (95% CI)	p value
ARPi agent	Abiraterone	Reference	-
	Enzalutamide	1.28 (0.81-2.01)	0.294
Age	Per 1-year increase	1.00 (0.97-1.03)	0.910
Baseline PSA	Log-transformed	0.95 (0.81-1.11)	0.503
Visceral metastasis	No	Reference	-
	Yes	1.01 (0.40-2.55)	0.991
Bone marrow involvement	No	Reference	-
	Yes	0.88 (0.41-1.88)	0.741
Number of metastatic sites	Per 1-site increase	1.11 (0.68-1.84)	0.673
Hemoglobin	Per 1 g/dL increase	0.79 (0.67-0.93)	0.004
ALP	Log-transformed	1.56 (1.04-2.35)	0.034
LDH	Log-transformed	2.39 (1.26-4.50)	0.007
Albumin	Per 1 g/dL increase	0.28 (0.15-0.51)	<0.001
TTCR	≥12 months	Reference	-
	<12 months	1.49 (0.90-2.46)	0.121
Gleason score	<8	Reference	-
	≥8	2.76 (1.53-4.98)	<0.001

Values are hazard ratios from a single multivariable Cox regression model including all variables shown. The analysis included 136 patients and 95 death events. Model discrimination: C-index=0.830. PSA, ALP, and LDH were entered as log-transformed variables. ARPi, androgen receptor pathway inhibitor; CI, confidence interval; HR, hazard ratio; TTCR, time to castration resistance; PSA, prostate-specific antigen; ALP, alkaline phosphatase; LDH, lactate dehydrogenase.
