# Supplementary material for: Enzalutamide Versus Abiraterone After Docetaxel in Metastatic Castration-Resistant Prostate Cancer: Real-World Outcomes and Exploratory Prognostic Stratification
Source: J Clin Med. 2026 Jun 21;15(12):4816. doi: 10.3390/jcm15124816 (PMC13300754; doi:10.3390/jcm15124816)
Supplement: Supplementary file 1 [file jcm-15-04816-s001.zip › Supplementary Table S3.pdf]

Table S3. Cox regression analysis of individual risk score components for overall survival.

Variable	Category / value	Patients, n (%)	Univariable HR (95% CI)	p value	Multivariable HR (95% CI)	p value
PIV	≤457.99	69 (50.7)	Reference	-	Reference	-
	>457.99	67 (49.3)	1.06 (0.71-1.60)	0.764	1.02 (0.68-1.53)	0.941
TTCR	≥12 months	65 (47.8)	Reference	-	Reference	-
	<12 months	71 (52.2)	2.27 (1.48-3.47)	<0.001	2.09 (1.35-3.23)	0.001
Hemoglobin	>12 g/dL	63 (46.3)	Reference	-	Reference	-
	≤12 g/dL	73 (53.7)	4.84 (3.04-7.71)	<0.001	4.20 (2.59-6.82)	<0.001
Gleason score	<8	43 (31.6)	Reference	-	Reference	-
	≥8	93 (68.4)	2.51 (1.55-4.08)	<0.001	1.66 (1.00-2.74)	0.048

Values are presented as n (%) unless otherwise indicated. The multivariable model included all four risk score components shown in the table. The analysis included 136 patients and 95 death events. Model discrimination: C-index=0.733. PIV, pan-immune-inflammation value; TTCR, time to castration resistance; CI, confidence interval; HR, hazard ratio.
